# The influence of AlN buffer layer on the growth of self-assembled GaN nanocolumns on graphene

**DOI:** 10.1038/s41598-019-55424-z

**Published:** 2020-01-21

**Authors:** Andreas Liudi Mulyo, Mohana K. Rajpalke, Per Erik Vullum, Helge Weman, Katsumi Kishino, Bjørn-Ove Fimland

**Affiliations:** 1 Department of Electronic Systems, Norwegian University of Science and Technology (NTNU), NO-7491 Trondheim, Norway; 2 Department of Engineering and Applied Sciences, Sophia University, 102-8554 Tokyo, Japan; 3 SINTEF Industry, NO-7465 Trondheim, Norway; 4 Sophia Nanotechnology Research Center, Sophia University, 102-8554 Tokyo, Japan; 5 Present Address: Microsoft Quantum Materials Lab, Niels Bohr Institute, University of Copenhagen, 2100 Copenhagen, Denmark; 6 Present Address: Sophia University, 102-8554 Tokyo, Japan

**Keywords:** Nanowires, Nanoscale materials, Graphene, Nanoscience and technology, Nanoscale materials, Nanowires, Semiconductors, Nanowires

## Abstract

GaN nanocolumns were synthesized on single-layer graphene via radio-frequency plasma-assisted molecular beam epitaxy, using a thin migration-enhanced epitaxy (MEE) AlN buffer layer as nucleation sites. Due to the weak nucleation on graphene, instead of an AlN thin-film we observe two distinguished AlN formations which affect the subsequent GaN nanocolumn growth: (i) AlN islands and (ii) AlN nanostructures grown along line defects (grain boundaries or wrinkles) of graphene. Structure (i) leads to the formation of vertical GaN nanocolumns regardless of the number of AlN MEE cycles, whereas (ii) can result in random orientation of the nanocolumns depending on the AlN morphology. Additionally, there is a limited amount of direct GaN nucleation on graphene, which induces non-vertical GaN nanocolumn growth. The GaN nanocolumn samples were characterized by means of scanning electron microscopy, transmission electron microscopy, high-resolution X-ray diffraction, room temperature micro-photoluminescence, and micro-Raman measurements. Surprisingly, the graphene with AlN buffer layer formed using less MEE cycles, thus resulting in lower AlN coverage, has a lower level of nitrogen plasma damage. The AlN buffer layer with lowest AlN coverage also provides the best result with respect to high-quality and vertically-aligned GaN nanocolumns.

## Introduction

Recent progresses in the growth of GaN thin-film on graphene^1–6^ increasingly justify the possibility for graphene to be a good alternative substrate over the available conventional substrates, for instance Si^7,8^, SiC^9^ and sapphire^10^. It is also reported that GaN thin-film is achievable on sapphire or amorphous substrates by employing either multi-layer or single-layer graphene as buffer layer^11–14^. Such exploitations can be realized by taking advantage of the weak *quasi*-van der Waals binding^15–17^ which can drastically relax the lattice matching condition to be satisfied by constituent materials. That being said, the lack of chemical reactivity of graphene and its extremely low surface energy^18^ greatly disrupt the nucleation process of GaN, resulting in a low nucleation density^19^ and difficulty in controlling the stacking sequence of the GaN growth^3^. The latter issue, which often manifests in the form of stacking faults^3,5^ or threading dislocations^1,4,17^, can possibly be suppressed by growing nanowire or nanocolumn structures^16,20–25^. Nevertheless, because of the absence of dangling bonds in graphene, nanocolumns are often aligned in non-vertical directions^26–28^ and/or have low density^27,29^. Both problems are required to be addressed in order to demonstrate that the growth of III-N semiconductor nanostructures, particularly nanocolumns, utilizing graphene as the substrate can be further considered as an alternative platform in enabling the advancement of optoelectronic device performance and functionality, for instance light-emitting diodes^30^.

In this regard, the introduction of defects in graphene by means of plasma treatments could alleviate the issue of absence of dangling bonds in graphene. In case of GaN grown by metal-organic vapor phase epitaxy (MOVPE), the creation of dangling bonds in graphene by this method has been achieved via *ex-situ* manner^4,14^. However, the plasma treatments to prepare the growth of GaN on graphene in radio-frequency plasma-assisted molecular beam epitaxy (RF-PAMBE), which is typically done *in-situ*, often lead to the generation of more severe plasma-induced damage of graphene^28,31^. Moreover, it has been evidenced that the GaN nanocolumns may be randomly oriented if the growth is done directly on graphene^27,28^. The use of a thin AlN buffer layer can provide substantial protection to the graphene from the direct bombardment of nitrogen plasma and enable the growth of high-density, vertically-aligned GaN nanocolumns on graphene^27,28^. Therefore, it is now perceived that the understanding of AlN as an intermediate layer in the GaN/graphene system is necessary to grasp the growth behavior of the GaN nanocolumns in relation to the graphene properties at a given growth condition. Furthermore, it is expected that the insight towards these aspects can shed light on the preference of certain growth conditions over others, which later can be utilized for growing device structures.

In the present work, we concentrate on the RF-PAMBE synthesis of self-assembled GaN nanocolumns on transferred single-layer graphene on silica glass for different AlN growth conditions. An insight towards the relation between the AlN migration-enhanced epitaxy (MEE) cycles and the morphology of the AlN buffer layer, as well as the resulting structural and optical properties of the grown GaN nanocolumns on graphene with AlN used as an intermediate layer, is reported. Additionally, the impact of the AlN buffer layer growth on graphene structural properties is presented.

## Results and Discussion

**Figure 1:**
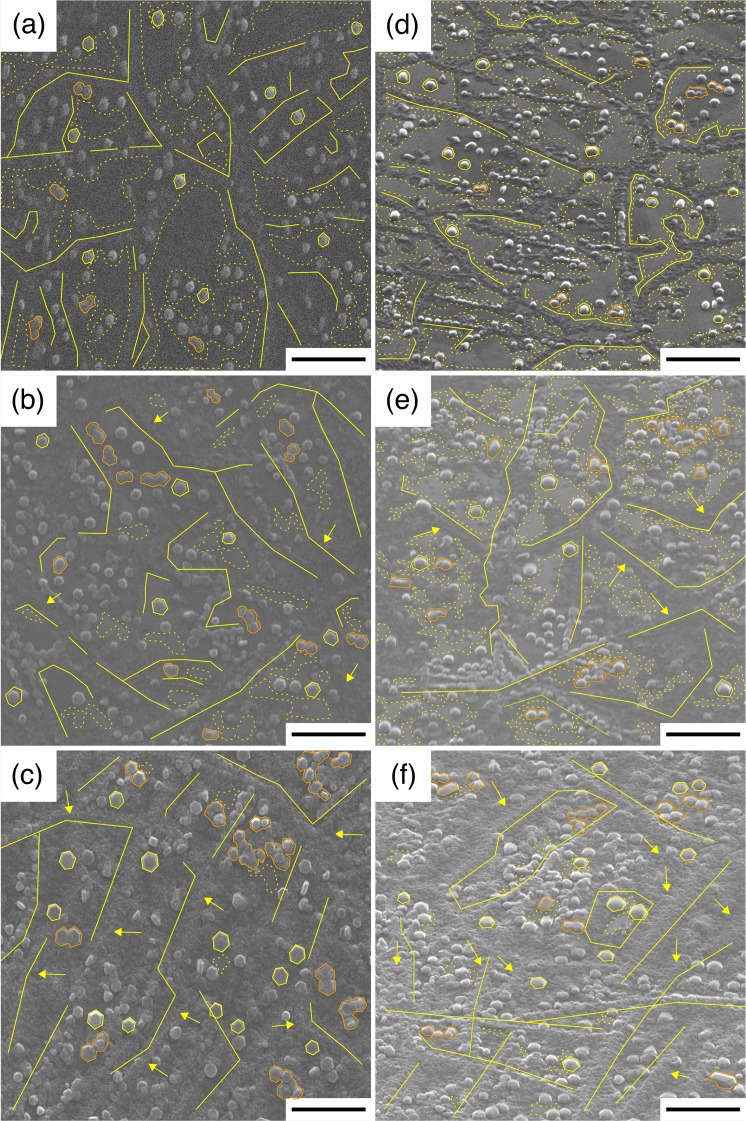
SEM images of AlN on graphene formed via different MEE cycles. (**a**,**d**), (**b**,**e**) and (**c**,**f**) are (top-, bird’s eye-view) SEM images of samples A1, A2 and A3, respectively. Scale bars are 1 μm. Features marked with yellow lines, yellow (orange) contours and yellow dashed outlines are high-density AlN nanostructures grown along line defects of graphene, individual (coalesced) AlN islands and areas of exposed graphene, respectively. Yellow arrows in samples A2 and A3 show the lateral growth of AlN nanostructures that initially nucleate at the line defects of graphene in sample A1.

The morphology of the thin AlN buffer layer formed on the graphene surface using MEE by RF-PAMBE technique was studied as a function of the amount of AlN deposited, defined by the number of MEE cycles. Details concerning the growth parameters for the AlN buffer layer are outlined in Methods. Three different AlN samples, namely A1, A2 and A3, with respectively 20, 40 and 80 MEE cycles, were grown and characterized. Figures 1(a,d),  (b,e) and (c,f) depict top- and bird’s eye-view scanning electron microscopy (SEM) images of samples A1, A2 and A3, respectively. In sample A1 there are still numerous areas of exposed graphene (marked with yellow dashed outline). These areas are reduced with the increasing number of MEE cycles, as shown in sample A2 and further A3. We note that there are two distinct arrangements of AlN on graphene. The *first feature* is the nucleation of high-density and irregular AlN nanostructures along the line defects of graphene (e.g., marked with the yellow lines), which is similar to the cases of AlGaN nucleation by MOVPE on graphene^29^. Commonly, line defects observed in chemical vapor deposition (CVD)-graphene are wrinkles and grain boundaries^32–34^. In addition to the surface morphology of the copper foil and the transfer process^34^, the wrinkles are often related with the compressive stress caused by the difference in thermal expansion coefficients between graphene and the copper foil during cooling from CVD growth temperature to room temperature (RT)^35^. The grain boundaries are formed as a result of coalesced graphene grains which are grown on the different copper facets during the CVD growth process^36^. These areas of graphene likely contain defects, which can eventually create dangling bonds to support covalent/chemical bonding of atoms, resulting in a high nucleation density of AlN nanostructures on those particular graphene sites. From bird’s eye-view SEM images, one clearly sees how the nucleation of AlN nanostructures spreads further away from the line defects with the increase of MEE cycles (e.g., marked with the yellow arrows). This will eventually cover the graphene surface, as shown in sample A2, and furthermore in sample A3.

The *second feature* is the formation of individual AlN islands (e.g., marked with the yellow contours). Unlike the high-density AlN nanostructures along the graphene line defects described above, their size are quite significant, with average diameters of approximately 70, 120 and 200 nm for samples A1, A2 and A3, respectively. In addition to the formation of individual AlN islands, it is also possible for the AlN islands to merge with their nearest vicinity to form coalesced AlN islands (e.g., marked with the orange contours). A tendency for AlN to form an island (and its cluster formation) instead of a thin-film could be caused by the extremely low surface energy (lack of dangling bonds/chemical inertness) of graphene^18^, which promotes the high surface tension on graphene surface. In addition, the lack of dangling bonds in these graphene surface areas hampers the AlN nucleation process to form a continuous thin-film structure, contrary to the formation of high-density AlN nanostructures in which the dangling bonds are provided by the defects along the graphene line defects. As opposed to this, when the MEE technique was applied to grow AlN on a sapphire substrate at a similar substrate temperature, it resulted in large island sizes and good coverage on the substrate surface^37^.

**Figure 2:**
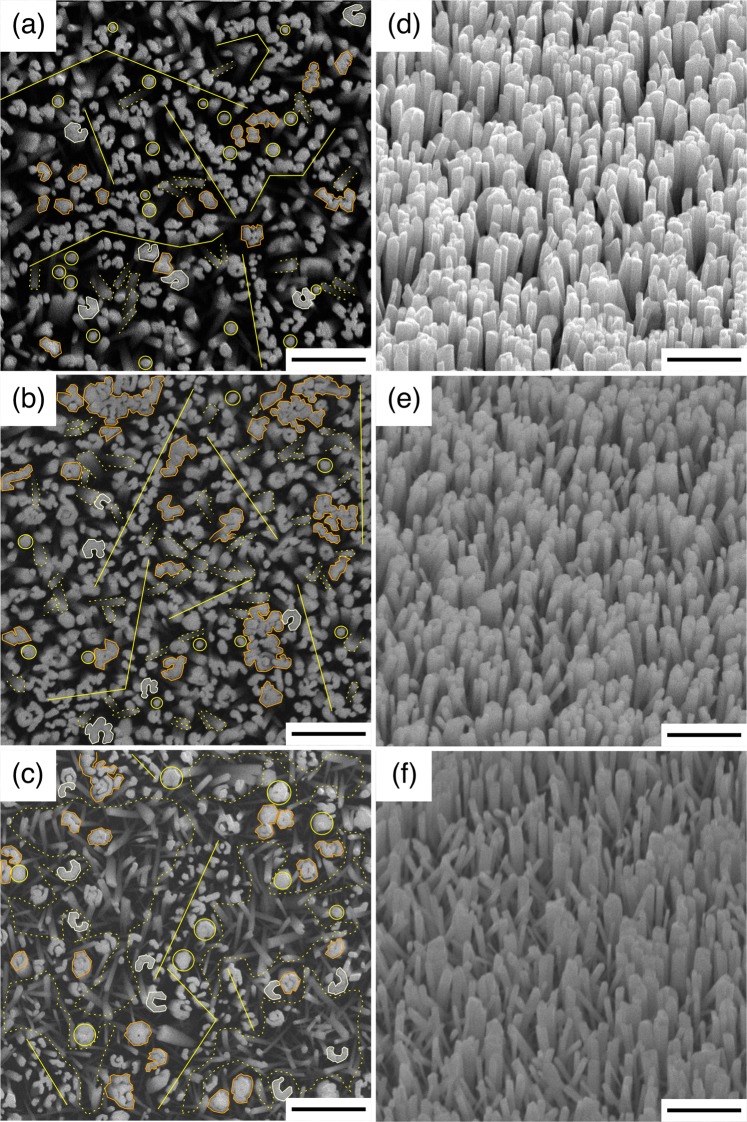
SEM images of GaN nanocolumns on graphene formed via different AlN MEE cycles. (**a**,**d**), (**b**,**e**) and (**c**,**f**) are (top-, bird’s eye-view) SEM images of samples G1, G2 and G3, respectively. Scale bars are 1 μm. Yellow lines, yellow circles (orange contours) and yellow dashed outlines indicate row of high-density vertical GaN nanocolumns, individual (coalesced) vertical GaN nanocolumns and areas with individual tilted GaN nanocolumns, respectively. White outlines in sample G3 indicate the GaN nanotubular-like structures.

Further RF-PAMBE growth of GaN nanocolumns was then carried out on samples A1, A2 and A3, after which they were re-named as G1, G2 and G3, respectively. In order to gain insight into the influence of the AlN buffer layer on the formation of GaN nanocolumns, the growth conditions of GaN nanocolumns on samples G1, G2 and G3 were kept the same (described in Methods). The representative top- and bird’s eye-view SEM images for samples G1, G2 and G3 are given in Fig. 2(a,d), (b,e) and  (c,f), respectively. We notice that the nanocolumns follow a particular arrangement which resembles the previously grown AlN buffer layer. The *first group of features* is the row of high-density vertical GaN nanocolumn growth (e.g., marked with the yellow lines), where nanocolumn diameters vary from 50 to 70 nm. More often, these nanocolumns are found to be grown very close to their neighbor, which is very likely to form coalesced nanocolumn structures, rather than single isolated nanocolumns. Such formation of coalesced GaN nanocolumn structures is clearly visible in sample G1. When formed on 40 MEE cycles AlN buffer (sample G2), it becomes more difficult to discern the rows of vertical GaN nanocolumns, since the density of the nanocolumn structures in general is increased. However, this unique pattern is still observable if one follows the GaN nanocolumns lining with each other, where the nanocolumns have a tendency to coalesce with their vicinity. Interestingly, GaN nanocolumns in sample G3, which has the highest number of AlN MEE cycles, do not meet the expectation of being the most coalesced nanocolumns, forming GaN nanowall-like structures, but rather display nanocolumn structures with identical diameters to those of sample G1.

**Figure 3:**
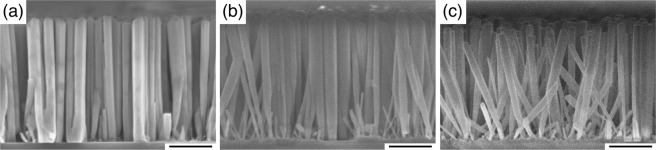
Cross-sectional SEM images of GaN nanocolumns on graphene formed via different AlN MEE cycles. Samples (**a**) G1, (**b**) G2, and (**c**) G3. Scale bars are 500 nm.

The *second group of features* is the individual vertical GaN nanocolumns (e.g., marked with the yellow circles). It appears that the number of MEE cycles during AlN formation affects the diameter of the grown nanocolumns, where less cycles give smaller diameter. It is found that the GaN nanocolumns in samples G1, G2 and G3 show average diameters of 90, 130 and 210 nm, respectively. Additionally, it is discovered that there are certain nanocolumns (e.g., marked with the orange contours) whose diameter is considerably larger than the mentioned average values. Some of them seem to result from single nanocolumn growth which eventually merges with other nanocolumns in its proximity forming an irregular shape, while there are also circular-shaped nanocolumns where the coalescence process likely starts immediately from the nucleation stage. It should be remarked that GaN nanotubular-like structures (white outlines) can also be formed^38^ because the edge of the AlN islands can initiate GaN nanocolumn growth^39^. The *third group of features* is the formation of tilted nanocolumns (see areas marked with yellow dashed outlines in Fig. 2) that have an average diameter of 70 nm for all three G-samples. Overall, samples G1 and G2 exhibit less tilted nanocolumns compared to sample G3, where tilted nanocolumns dominate over the vertical ones. Representative cross-sectional SEM images of samples G1, G2 and G3 in Fig. 3(a–c), respectively, also show this. The average length (height) of the vertical GaN nanocolumns (first and second group) is around 1400 nm, while the length of tilted nanocolumns (third group) varies from 175 to 1400 nm. The large variation noticed in the latter group of nanocolumns is possibly caused by a shadowing effect during the GaN growth. With increased number of MEE cycles, the AlN surface turns rougher and the tilted nanocolumns in the G-samples become more discernible.

**Figure 4:**
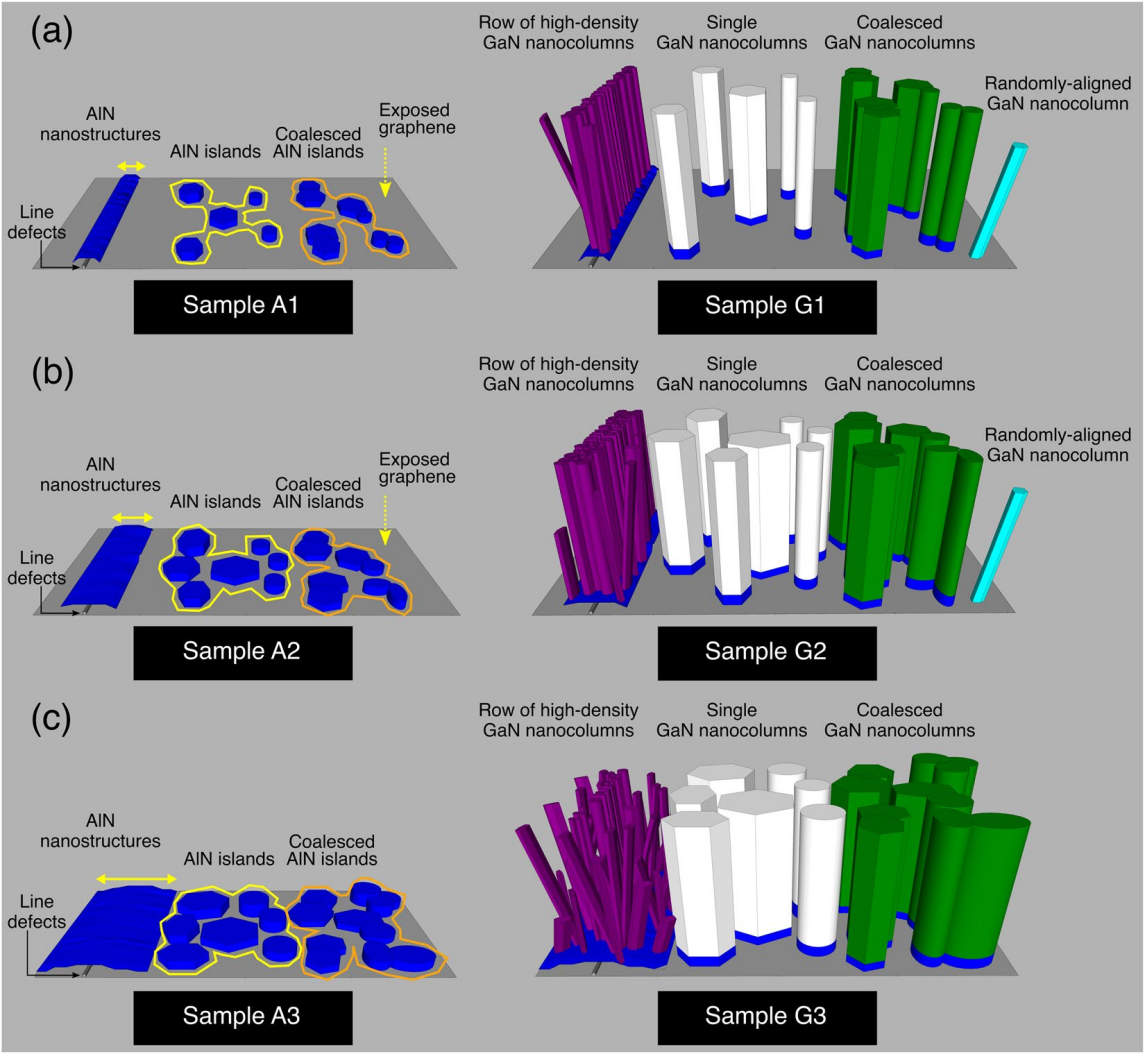
Simplified schematics of the AlN buffer structures and GaN nanocolumn formation on graphene. Samples (**a**) A1-G1, (**b**) A2-G2 and (**c**) A3-G3. There are two possible AlN (blue) nanostructures forming on graphene: 1) AlN islands and 2) AlN nanostructures along the line defects of graphene (the yellow arrows indicate their lateral growth spread further away from the line defects). Single (white) and coalesced (green) vertical GaN nanocolumn structures are nucleated from the AlN islands, while row of high-density nanocolumns (purple) form on the AlN nanostructures that spread from the line defects. Additional tilted nanocolumns (cyan) are likely to grow on exposed graphene.

Based on the SEM observations in Figs. 1–3, an illustrative representation of the AlN buffer layer and GaN nanocolumn growth on graphene is presented in Fig. 4. The MEE growth of an AlN buffer layer in sample A1 results in the formation of AlN islands and a high density of AlN nanostructures (growing along the graphene line defects). A higher number of MEE cycles generally increases the lateral size and the density of these AlN features, which could lead to coalesced structures. This will eventually reduce the area of exposed graphene, until it is finally almost absent in sample A3 (Fig. 1(c,f)). For the subsequent GaN growth, it is likely that the single vertical GaN nanocolumn structures (indicated by white color in Fig. 4) nucleated on the AlN islands, as the average GaN nanocolumn diameter is comparable to that of the AlN islands. The same applies to those grown on the coalesced AlN islands, resulting in coalesced nanocolumns (indicated by green color in Fig. 4). Next, the row of high-density GaN nanocolumns (indicated by purple color in Fig. 4) most likely forms on the high-density AlN nanostructures. Vertical nanocolumns are expected to form here, however it is possible to get tilted ones as well due to the misalignment of the AlN lattice formed on defective graphene areas^40^, including graphene line defects. The latter becomes more pronounced with a higher number of AlN MEE cycles, especially in sample G3 (Fig. 4(c)). Indeed, randomly-aligned nanocolumn growth could be also due to limited direct nucleation of GaN on exposed graphene (indicated by the cyan color in Fig. 4(a,b)), however this is not likely for sample G3 since the AlN covers nearly the whole graphene surface. It might be that the rough surface (many crystallites of irregular shape) of AlN nanostructures, which are formed near (but away) from the graphene line defects induces the deterioration of GaN nanocolumn growth orientation.

**Figure 5:**
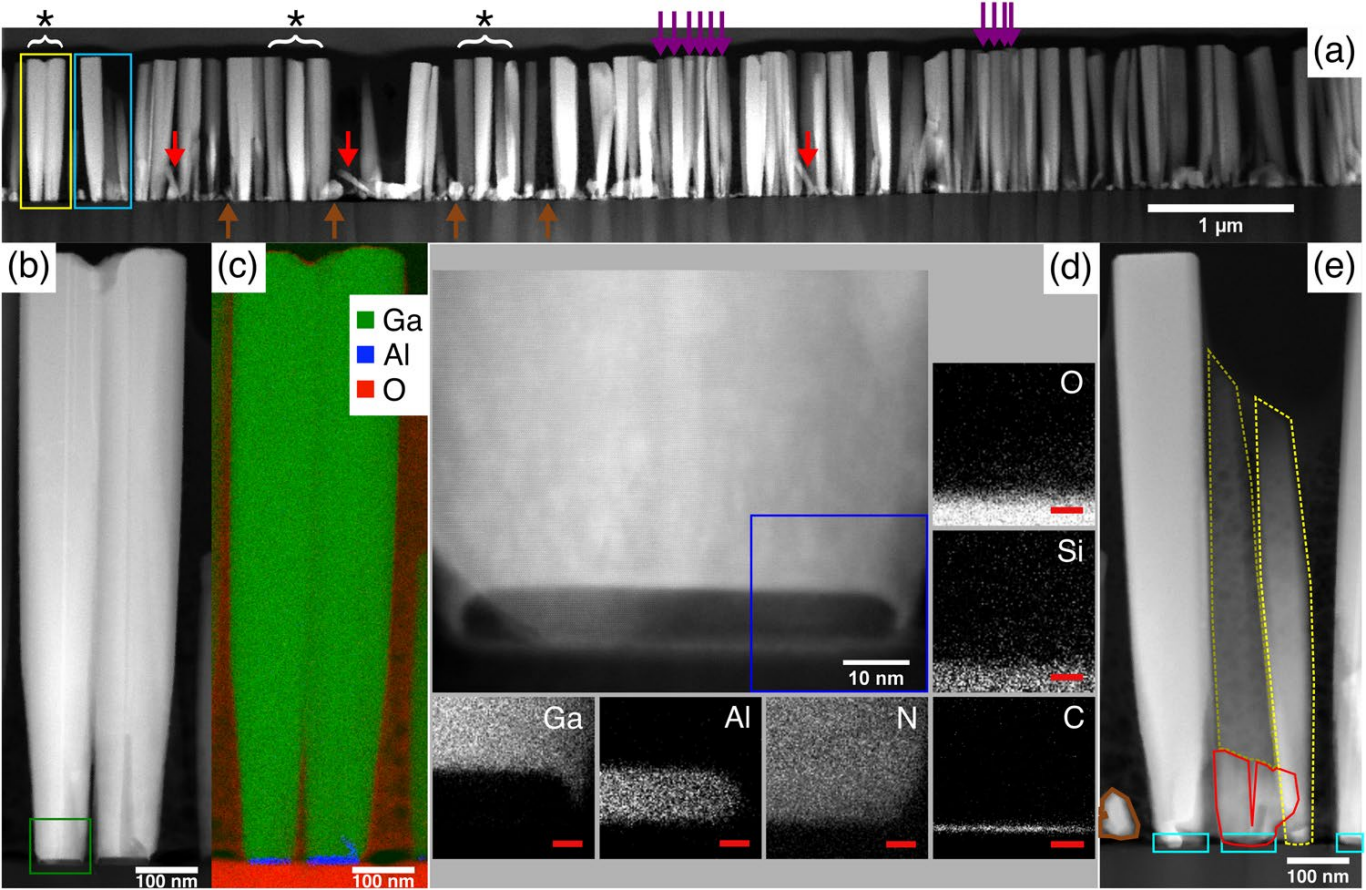
TEM image of GaN nanocolumn sample synthesized with nominally the same growth conditions as sample G1. (**a**) Overview cross-sectional HAADF STEM image, showing vertical GaN nanocolumns (star-marked and purple arrows), inclined GaN nanocolumns (red arrows) and GaN crystallites (brown arrows). (**b**) HAADF STEM image of two GaN nanocolumns within yellow frame in a. (**c**) Combined color map of the Ga (green), Al (blue) and O (red) elemental distributions (EDS/EELS) on the corresponding region in b. (**d**) Magnified image of the lower part of the GaN nanocolumn near the interface of the left GaN nanocolumn in b (green frame), with the elemental mapping near the interfaces between the GaN nanocolumn, AlN island, graphene and silica glass (blue frame) by EDS (Ga, Al, Si) and EELS (N, O, C). The red scale bars are 5 nm. (**e**) HAADF STEM image of the GaN nanocolumns that is light-blue framed in a. The inclined GaN nanocolumn (yellow-dashed line) is possibly directly nucleated on graphene (indicated by the absence of any AlN layer [cyan frames] at the base). There are two broken GaN nanocolumns (red framed area) sharing the same AlN island and another inclined GaN nanocolumn (dark-yellow dashed line) in the background. An irregular GaN crystallite (brown outline) likely grown directly on graphene is also observed.

A separate nanocolumn sample was synthesized with nominally the same growth conditions as sample G1 for transmission electron microscopy (TEM) measurements. The cross-sectional high-angle annular dark field scanning TEM (HAADF STEM) image of Fig. 5(a) depicts that most of the observed nanocolumns exhibit a perpendicular growth direction with respect to the substrate (exemplified by star-marked and purple arrows). The average height of the nanocolumns is 400 nm shorter than for the nanocolumns in sample G1. This difference could be caused by a reduced growth rate (after chamber maintenance) during the growth of the sample for the TEM measurements. Additionally, the image reveals irregular GaN nanostructures like short inclined GaN nanocolumns (red arrows) and GaN islands/crystallites (brown arrows). Two GaN nanocolumns with star symbol (yellow frame in Fig. 5(a)) are closely examined in Fig. 5(b). At the base there is an area of darker contrast, attributed to AlN owing to the atomic number difference between Al and Ga (the intensity contrast in HAADF STEM images scales approximately with Z^2^, where Z = the atomic number). Simultaneous energy dispersive spectroscopy (EDS) and electron energy-loss spectroscopy (EELS) were carried out on the same area to map all present elements. The Ga, Al and O element maps are combined into a color map in Fig. 5(c), which clearly shows that Al (blue) appears at the base of nanocolumns (with a measured height and diameter of ~10 and ~65 nm, respectively), and Ga (green) is identified at the nanocolumn regions. Interestingly, no trace of any Al signal is detected in between the GaN nanocolumns or in the region outside the area where the vertical GaN nanocolumns are successfully synthesized. O (red) is detected in the silica glass substrate, as expected. Before this discussion is continued, we would like to point out that the vertical GaN nanocolumns marked by purple arrows in Fig. 5(a) have a top diameter of 50–70 nm, which is smaller than for the GaN nanocolumns marked with star symbol (average diameter of 90 nm). Based on the SEM observations explained in Fig. 2(a), there is a high probability that this group of GaN nanocolumns is grown on AlN nanostructures on or near line defects of the graphene.

A higher magnification of the GaN nanocolumn interface area (green frame in Fig. 5(b)) is presented in Fig. 5(d) together with six different element maps (Ga, Al, N, C, Si and O) of the blue framed area. Here, one can clearly distinguish that the AlN island acts as a nucleation site for the GaN nanocolumn. Three things to note from the elemental mapping observation: 1) A sharp interface separates Ga in the GaN nanocolumn and Al in the AlN island with no severe intermixing between them; 2) N is distributed evenly in the GaN nanocolumn regions and AlN island; 3) A distinct signal from C, implying the existence of graphene between the silica glass (Si, O) and the AlN island. Figure 5(e) presents a close-up image from the light-blue framed area in Fig. 5(a). Whereas intensity contrasts reveal the presence of AlN islands within the cyan frames, no indication of an AlN island is observed at the base of the inclined GaN nanocolumn marked with a yellow-dashed line. Moreover, its diameter of 50 nm is similar to the dimensions of the tilted GaN nanocolumns described in Fig. 2. Next to this tilted GaN nanocolumn, there are broken parts of other GaN nanocolumns (red frame) which are nucleated on the same AlN island. The GaN nanocolumn that appears to be on top of these parts (dark-yellow dashed line) is a different GaN nanocolumn located in the background. Notice as well the irregular GaN crystallite to the left (brown outline) which also does not have AlN underneath it. In similar fashion, other inclined GaN nanocolumns (red arrows) and GaN crystallites (brown arrows) shown in Fig. 5(a) could have had their nucleation occurring directly on the graphene.

**Figure 6:**
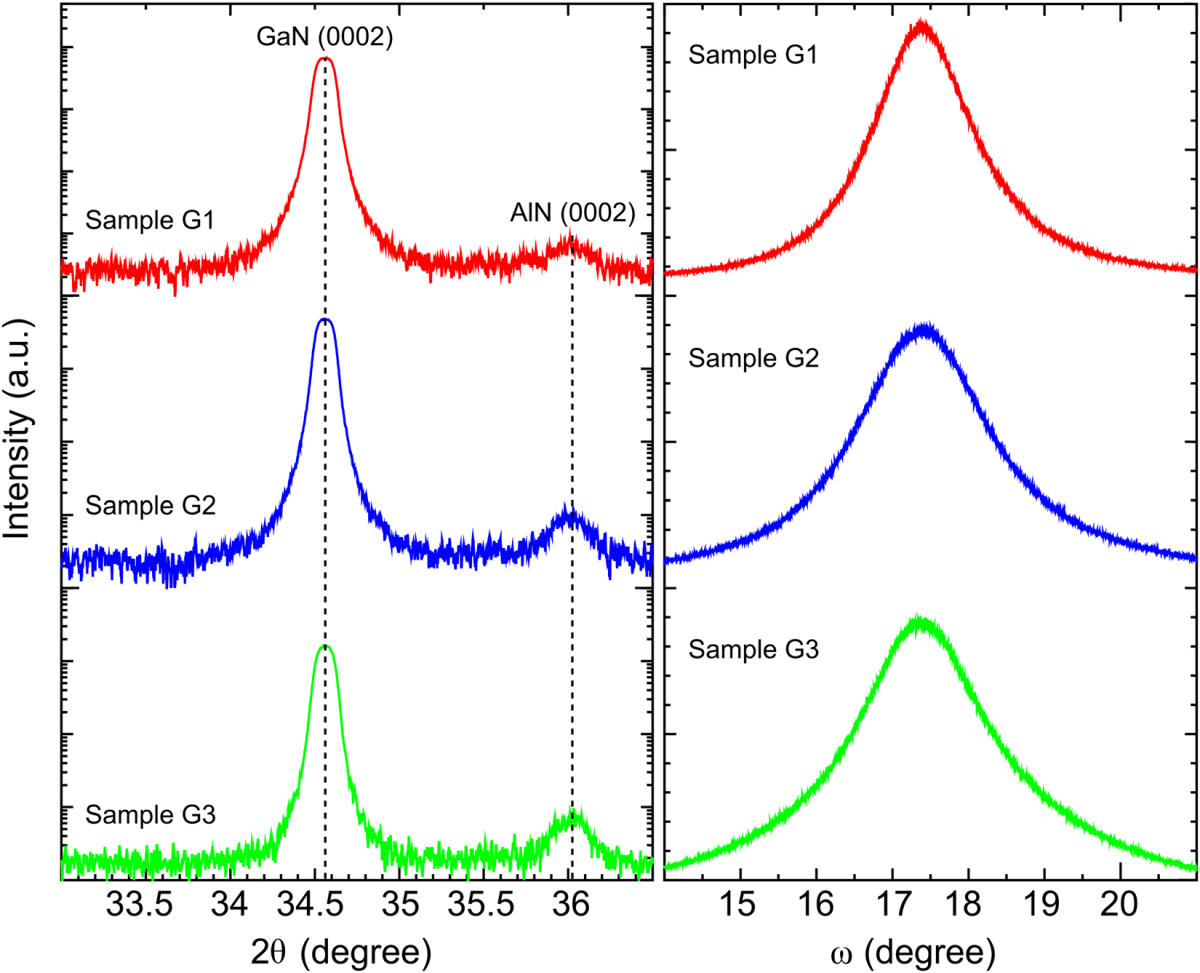
HRXRD measurements of the nanocolumns. (**a**) 2θ-ω scanning curve and (**b**) ω scanning curve of samples G1, G2 and G3.

From the intense peak observed at 34.56° in the 2θ-ω high-resolution X-ray diffraction (HRXRD) scans presented in Fig. 6(a), it is confirmed that all the GaN nanocolumn samples demonstrate growth orientation along the *c*-axis of the wurtzite GaN structure. Aside from this peak, a weak peak detected at ~36° is assigned to wurtzite AlN with the same crystal orientation as the GaN. Despite of these similarities, we find that the AlN buffer layer formed using different number of MEE cycles influences the GaN signals. The peak intensity of the 0002 diffraction of GaN is found to be highest for sample G1 and lowest for sample G3. Since this peak only represents signal from the planes normal to the *c*-axis (i.e. the 0002 reflection) of the GaN crystal, it indicates that sample G1 with 20 AlN MEE cycles has the highest density of vertical nanocolumns (since the SEM characterization presented above show that G1 has the lowest average GaN nanocolumn diameter [90 nm] among the G-samples). We note that whereas the peak intensity of the AlN 0002 reflection is higher for sample G2 than for sample G1, there is not much difference in this peak between sample G2 and sample G3, despite higher number of AlN MEE cycles for sample G3 and improved graphene coverage surface area (Fig. 1). It is possibly mostly reflection from AlN islands that contributes significantly to this peak, since the crystalline quality of AlN nanostructures grown on graphene defects is most likely polycrystalline^40^. (We should here point out that the 2θ-ω scans presented in Fig. 6(a) were recorded with a wider receiving slit than the normal [0.6 mm vs. 0.1 mm, respectively], in order to record the weak signal from the AlN buffer. Using the wider slit causes the peaks to be slightly broadened, as is seen by the “rounded out” top of the peak.)

The ω (rocking) scans of the GaN, shown in Fig. 6(b) with peaks centered at 17.28°, reveal that the full-width at half-maximum (FWHM) broadens with the higher number of AlN MEE cycles, increasing from 1.6 to 2.3 and 2.4° for samples G1, G2 and G3, respectively. The larger FWHM shown by the GaN nanocolumns in samples G2 and G3 relative to G1 indicates poorer orientation, i.e. larger GaN nanocolumn tilt distribution in samples G2 and G3. (0.1 mm receiving slit width was used for recording the rocking curves presented in Fig. 6(b)).

**Figure 7:**
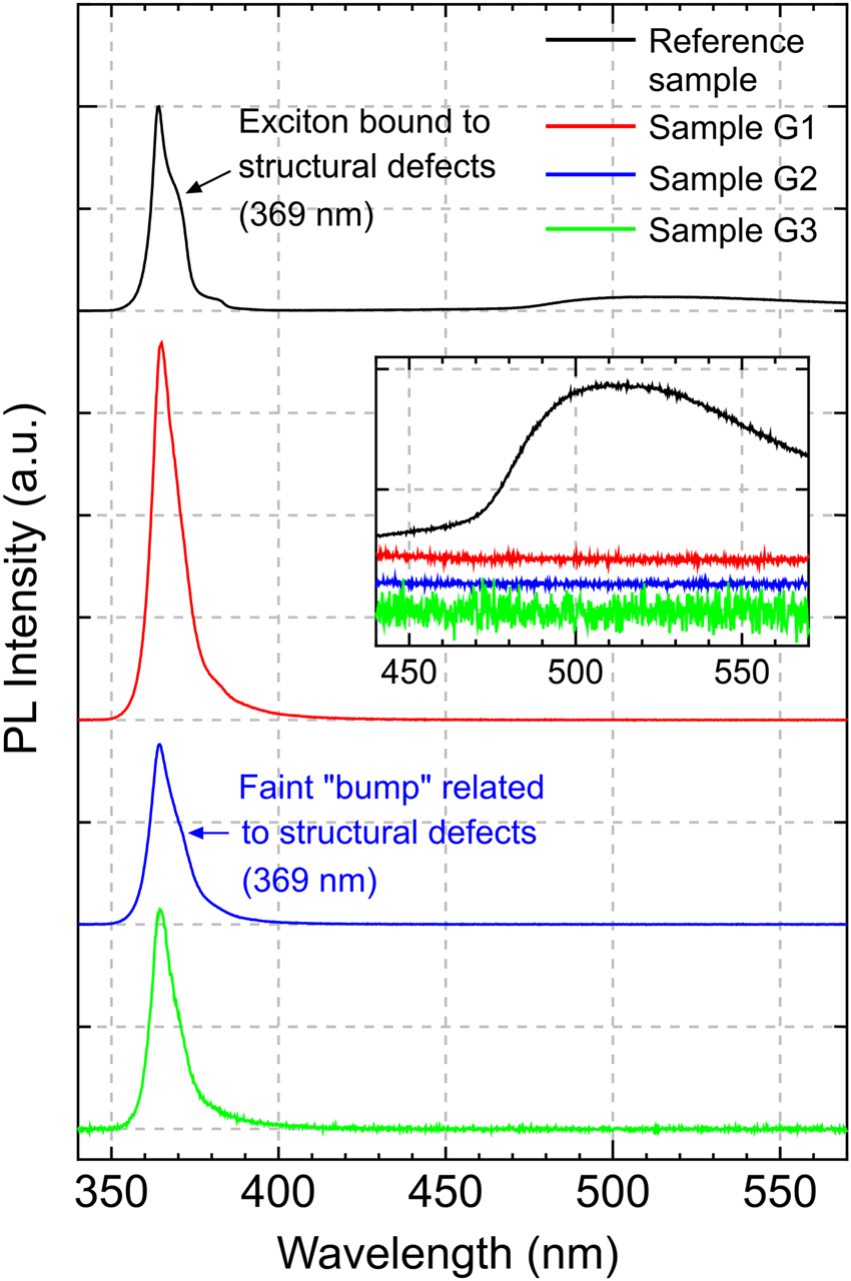
RT micro-photoluminescence spectra of reference sample (HVPE-freestanding GaN), samples G1, G2 and G3. Inset shows the magnified spectra in the wavelength range from 440 to 570 nm, highlighting the presence of broad green and yellow emission band in the reference sample.

Room temperature micro-photoluminescence measurements from the reference sample, samples G1, G2 and G3 clearly show distinguished emission peaks at 363.6, 364.6, 364.5 and 364.5 nm, respectively (Fig. 7), and can be associated with wurtzite GaN. In terms of linewidths, GaN nanocolumns in samples G1, G2 and G3 demonstrate narrower FWHM compared to the freestanding hydride vapor-phase epitaxy (HVPE)-GaN bulk substrate reference sample, being respectively 9.02, 10.17 and 8.62 nm, against 10.79 nm. The peak intensity of the GaN near band-edge emission among the GaN nanocolumn samples is highest for sample G1, which is almost two times more intense than that of the reference sample. Unlike for sample G1, emissions of GaN nanocolumns in samples G2 and G3 exhibit approximately similar peak intensity with the reference sample. Such unexpected low emission peak intensity from the GaN nanocolumn structure in these two samples is possibly caused by two separate driving factors. GaN nanocolumns in sample G2 (Fig. 2(b)) are shown to have a higher degree of coalescence relative to that of samples G1 and G3 (Fig. 2(a,c)), which can lead to the degradation of crystalline quality via formation of defects or dislocations^24^. For sample G3, the grown GaN nanocolumns characterized with lower density (Fig. 2(c)), and shorter nanocolumn height compared to sample G1 (Fig. 3(c)), thus inducing smaller excitation volume^41^, could potentially reduce the photoluminescence intensity.

None of the G-samples show any emission at 386 nm which is related to the GaN zinc blende crystal phase^3,42^. Unlike the reference sample, the GaN nanocolumn samples G1 and G3 do not display any shoulder peak at 369 nm, which is attributed to excitons bound to structural defects^43^. Apparently there is a faint “bump” in the spectrum of sample G2 related to dislocation-related emission, which is likely correlated with the higher defect density due to more nanocolumns being coalesced as to that of nanocolumns formed in samples G1 and G3. The distinct presence of green and yellow luminescence bands^44^ in the reference sample covering the wavelengths from 465 to 570 nm (emphasized in the inset of Fig. 7) is associated to isolated defects involving Ga vacancies and to the same defect bound to structural defects^45^. The absence of these broad bands in the GaN nanocolumn samples can also be an indication that the grown nanocolumns are free from threading edge dislocations^46^.

**Figure 8:**
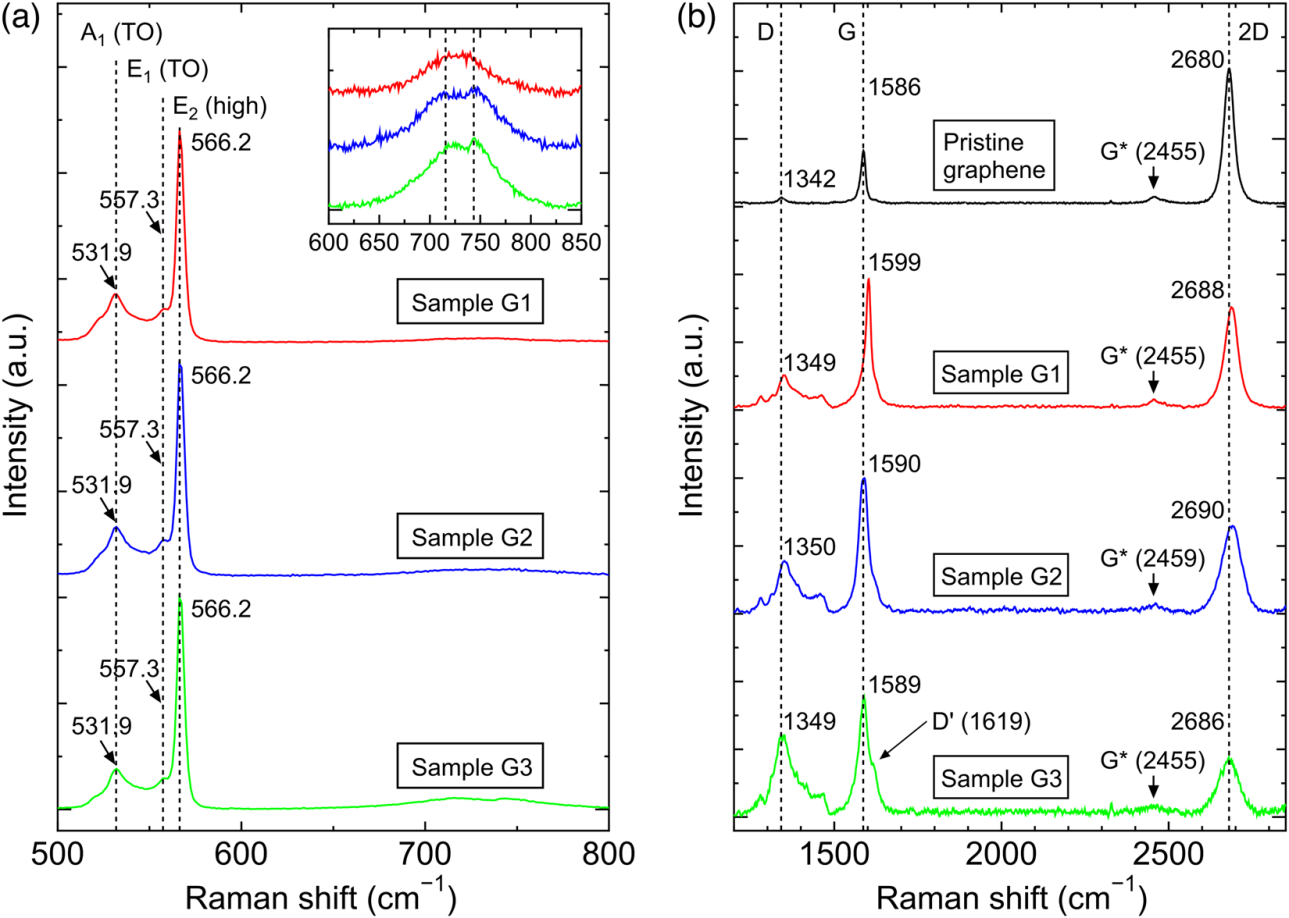
Micro-Raman spectroscopy of the nanocolumn samples, including the graphene for each respective sample. Raman spectra of (**a**) samples G1, G2 and G3 between 500 and 800 cm^−1^, with the peak frequencies of the A_1_ (TO), E_1_ (TO) and E_2_ (high) modes indicated by vertical dashed lines (inset: magnification from 600 to 850 cm^−1^, with the identified peak frequencies at 715 and 743 cm^−1^ of the possible SO and LPP modes, respectively^48,52^, indicated by vertical dashed lines), and (**b**) pristine graphene, samples G1, G2 and G3 between 1100 and 3200 cm^−1^. The dashed lines indicate D, G and 2D peak positions of pristine graphene.

Raman spectra of previously studied GaN nanocolumns, either grown in self-assembled manner with an AlN buffer layer on sapphire or Si substrates^47,48^ or synthesized selectively using a Ti mask on GaN template/sapphire^49^_,_ demonstrate features at (531.5 ± 0.5), (557.9 ± 0.9) and (566 ± 1.0) cm^−1^. These wave numbers correspond well to the established A_1_ (transverse optical (TO)), E_1_ (TO) and E_2_ (high) phonon modes of wurtzite GaN, respectively^50–53^. In line with the former results, the as-grown GaN nanocolumns in samples G1, G2 and G3 likewise exhibit almost identical spectra. As presented in Fig. 8(a), they show the peaks at 531.9, 557.3 and 566.2 cm^−1^. In addition, the E_2_ (low) phonon mode (not shown in Fig. 8(a)) is observed at 144 cm^−1^^48,50^. Notice that the identified peak of E_2_ (high) mode at 566.2 cm^−1^ and its small FWHM (~4.9 cm^−1^, comparable to that of GaN nanocolumns on Si(001) and Si(111)^54^) correspond to the FWHM of a fully relaxed thick free-standing GaN layer^55^, which can be an indicator of the high crystalline quality of the GaN nanocolumns. Furthermore, the TO phonon mode at 555 cm^−1^ related to zinc blende GaN^50,51^ is not detected in any of the GaN nanocolumn samples. However, expected A_1_ (longitudinal optical, LO) and E_1_ (LO) phonon modes of GaN^50,51^ at 734 and 741 cm^−1^ are not visible in any of the GaN nanocolumn samples, similar to the results obtained by Robins *et al*.^48^ and Jeganathan *et al*.^52^. Instead, we notice very weak peaks at 715 cm^−1^ and 743 cm^−1^ (inset in Fig. 8(a)) which might be related to the existence of surface optical (SO) and single LO or longitudinal phonon-plasmon (LPP) modes, respectively, whose frequencies and line widths are dependent on GaN carrier concentrations^48,52,53^. Further study is required to confirm this phenomenon.

In comparison with the pristine graphene reference sample (i.e. sample before any III-V growth), graphene structural properties after the GaN nanocolumn growth demonstrate quite significant alterations, as can be seen in Fig. 8(b). A typical Raman spectrum of the pristine graphene^56^ is strongly characterized with two main peaks, namely the G band (1586 cm^−1^) and the 2D band (2680 cm^−1^) with an intensity ratio of *I*_*2D*_*/I*_*G*_ ≈ 2, while the disorder-induced D band (1342 cm^−1^) is barely seen, making the intensity ratio of *I*_*D*_*/I*_*G*_ almost negligible. Changes to the aforementioned intensity ratios, peak positions and possible generation of another band are observed in all of the GaN nanocolumn samples (whereas a faint G* band at ~2456 cm^−1^ is unaffected). Firstly, the most prominent changes are the decline of the *I*_*2D*_*/I*_*G*_ ratio along with an increasing *I*_*D*_*/I*_*G*_ ratio from sample G1 to G2 and G3, implying that graphene becomes more defective as the number of AlN MEE cycles increases from 20 to 80. Secondly, another defect-related signature in graphene at 1619 cm^−1^, known as D’ band^56^, is noticed only in sample G3. Finally, the peak positions of the D, G and 2D bands experience slight blue shifts, indicating a compressive strain and/or a modification of the carbon atoms in the graphene network by the incorporation of nitrogen atoms which could lead to an *n*-type doping^57^. Such blue-shifting peaks have been observed when an intentional plasma nitridation of graphene using either nitrogen^58^ or ammonia^59^ causes the introduction of defects as well as changes in the chemical and electronic properties of graphene. Indeed, in this work such nitridation process is an inevitable part of the GaN nanocolumn growth in RF-PAMBE, although during the AlN buffer layer formation the exposure of nitrogen plasma is intended for the nitridation of Al. As shown in Fig. 1, one can evidently notice that the deposited Al could not form a continuous layer, particularly at lower numbers of MEE cycles. Thus, we cannot rule out the possibility of graphene being nitrided although it only occurs for three seconds per MEE cycle. With that in mind, it can be assumed that the higher number of MEE cycles could extend the duration of graphene being exposed to nitridation process.

## Conclusion

From the growth observation on the synthesis of GaN nanocolumns on graphene utilizing AlN buffer layers grown via MEE technique with 20, 40 and 80 cycles, it can be suggested that their morphology and orientation are restrained by the AlN structures on graphene. The high surface tension of graphene creates AlN islands on the graphene surface, beneficial for nucleation of GaN nanocolumns. Along with the islands, AlN nanostructures are densely formed along line defects (grain boundaries or wrinkles) of graphene due to the existence of defects which provides dangling bonds. Vertical GaN nanocolumns can be consistently obtained from the AlN islands independent of the number of AlN MEE cycles, whereas the GaN nanocolumns formed on AlN nanostructures can be randomly oriented when they are grown with higher number of AlN MEE cycles. There is also a likelihood of limited direct growth of GaN nanocolumns on graphene, resulting most likely in random growth orientations. Although higher number of MEE cycles produce wider AlN coverage on graphene, the subsequent GaN nanocolumns as well as the graphene property turn out to be not ideal. The structural characterizations and optical measurements present that GaN nanocolumn formation utilizing AlN buffer layer formed with 20 MEE cycles is the most preferable growth condition to obtain overall good quality and vertical alignment of GaN nanocolumns while maintaining the least damage on graphene.

## Methods

### Radio-frequency plasma-assisted molecular beam epitaxial growth

Commercially available 10 × 10 mm^2^ CVD-graphene grown on copper foil^34^ and transferred onto the center of a 2-inch supporting silica glass wafer (thickness of 0.5 mm) by Graphene Platform Corp. (Tokyo, Japan) was used as growth substrates. All the graphene substrates used in this work refer to an atomic layer of *sp*^2^ hybridized carbon atoms arranged in a hexagonal structure with an approximate thickness of 0.335 nm. The samples were grown by an EpiQuest RF-PAMBE system (at Sophia University) equipped with an RF nitrogen plasma source to generate active nitrogen and standard solid-source effusion cells (Knudsen cells) to provide Al, Ga and Si atoms.

In general, the growth was started by depositing the AlN buffer layer using the MEE technique^37,60^ at 805 °C, with expectation that the migration length of the supplied atoms on the substrate surface becomes longer than when using the conventional MBE method. Stated substrate temperatures were measured by a pyrometer [ref. 21]. MEE was executed by supplying Al and N atoms alternately to the graphene surface using an Al flux of 8.0 × 10^–5^ Pa, a N_2_ flow rate of 2.0 sccm and an RF power of 450 W. Each MEE cycle (or MEE period) of AlN consists of four seconds with Al atom supply, five seconds of interruption (resting time) and three seconds with N atom supply^27^. To investigate the effect of the AlN buffer layer, we grew AlN buffer layer samples A1, A2 and A3 with different layer thicknesses by employing 20, 40 and 80 MEE cycles, respectively. After SEM characterization, these samples were re-loaded into the RF-PAMBE growth chamber and further growth of GaN nanocolumns was performed with conventional MBE method using the following growth parameters^28^: a Ga flux of 2.5 × 10^−4^ Pa and a N_2_ flow rate of 2.75 sccm (RF power of 450 W) at a substrate temperature of 895 °C with a growth duration of 90 minutes. After the growth of GaN nanocolumns on samples A1, A2 and A3, the samples were re-labeled as G1, G2 and G3, respectively.

### Scanning electron microscopy

SEM images were obtained using an SII SMI3050SE focused ion beam-SEM and a Hitachi SU8000 SEM at acceleration voltages of 15 and 10 kV, respectively.

### Transmission electron microscopy

The interface of nanocolumn/buffer layer/graphene/silica glass and their element distribution were studied by high-resolution TEM and STEM-EELS in a double Cs-corrected cold field emission gun JEOL ARM200F operating at 200 kV, equipped with a QuantumER GIF for EELS. Details on the TEM specimen preparation can be found in ref. 28.

### X-ray diffraction

HRXRD was performed with a Bruker D8 Discovery High-Resolution Diffractometer using Cu Kα radiation (1.5406 Å).

### RT micro-photoluminescence measurements

Optical studies were done using RT micro-photoluminescence with a He-Cd laser (325 nm) as the excitation source. A freestanding HVPE-grown GaN bulk substrate (threading dislocation density of 6–8 × 10^6^ cm^−2^) was used as the reference sample like for previous studies^21,24^.

### Micro-Raman spectroscopy

Unpolarized micro-Raman spectroscopy was conducted at RT in backscattering configuration using a Renishaw InVia Reflex Spectrometer System equipped with a 514.5 nm excitation laser. A pristine graphene transferred onto silica glass was utilized as a comparison to graphene samples after RF-PAMBE growth.
